# Human sperm vitrification: the state of the art

**DOI:** 10.1186/s12958-020-00580-5

**Published:** 2020-03-07

**Authors:** Yong Tao, Erika Sanger, Arpornrad Saewu, Marie-Claude Leveille

**Affiliations:** Ottawa Fertility Center, 100-955 Green Valley Crescent, Ottawa, ON K2C 3V4 Canada

**Keywords:** Cryopreservation, Vitrification, Contamination, Liquid nitrogen, Spermatozoa, Semen

## Abstract

Sperm cryopreservation has been widely used in assisted reproductive technology (ART) and has resulted in millions of live births. Two principal approaches have been adopted: conventional (slow) freezing and vitrification. As a traditional technique, slow freezing has been successfully employed and widely used at ART clinics whereas the latter, a process to solidify liquid into an amorphous or glassy state, may become a faster alternative method of sperm cryopreservation with significant benefits in regard to simple equipment and applicability to fertility centers. Sperm vitrification has its own limitations. Firstly, small volume of load is usually plunged to liquid nitrogen to achieve high cooling rate, which makes large volume sample cryopreservation less feasible. Secondly, direct contact with liquid nitrogen increases the potential risk of contamination. Recently, new carriers have been developed to facilitate improved control over the volume and speed, and new strategies have been implemented to minimize the contamination risk. In summary, although sperm vitrification has not yet been applied in routine sperm cryopreservation, its potential as a standard procedure is growing.

## Background

Over 8 decades ago, in 1938, Luyet and Hoddap performed the first vitrification of frog sperm in liquid air [1]. Polge et al. [2] at the National Institute for Medical Research in London, England claimed “Revival of spermatozoa” after they successfully froze sperm samples from various species with glycerol [2]. Sperm cryopreservation has been widely used in assisted reproductive technology (ART) programs, including infertility treatment and cancer patient fertility preservation. Its application can be a therapeutic solution for patients with male infertility. The first attempt using vitrified sperm for human live birth was reported in 1953 by two researchers from State University of Iowa, using sperm frozen on dry ice [3], and soon after the publication, normal live birth was declared. About one decade later, the same group tried with liquid nitrogen (LN_2_) and succeeded [4, 5]. Live births from frozen sperm after 4 decades storage in LN_2_ had been reported [6]. Cryopreservation of spermatozoa has been the most valuable and used way to preserve the fertility of males, including those undergoing chemotherapy or radiotherapy, those with severe oligospermia or ejaculatory disorder, and those with disorders leading to testicular damage.

Currently, there are two principal methods applied to the cryopreservation of human sperm, namely, conventional freezing and vitrification. Conventional freezing, slow freezing, a traditional technique, has been successfully employed for the cryopreservation of human sperm many times in the past. In contrast, vitrification is a novel technique, and has been a quickly growing alternative method of rapid freezing.

Traditional cryopreservation techniques are still widely used at ART clinics all over the world, but vitrification has become more effective for cryopreserving human spermatozoa after the efforts in past decade [7]. Vitrification, by directly plunging the sperm samples into LN_2_, is a fast, simple and cost-effective method to cryopreserve human spermatozoa. This method of rapid freezing causes no damage from intracellular ice crystallization during cooling. Live births were achieved with vitrified spermatozoa by intracytoplasmic sperm injection (ICSI) [8] and by intrauterine insemination [9]. A healthy birth using a vitrified sperm sample was recently reported in Spain after a Day 5 blastocyst was monitored and transferred [10].

Cryoinjury, also called cryodamage, is the damage of cryopreserved biological materials due to water phase changes at low temperatures. The mechanisms involve osmotic rupture by extra- or intra-cellular ice formation. Cell viability largely depends on the integrity of plasma membrane as well as the cellular organelles inside [11]. The cooling velocities also affect the physicochemical and biophysical reactions, and thus alter the survival. The emergence of proteomics and transcriptomics may provide some inspiration to understand cryoinjury mechanisms [12–14].

Another perspective was raised to elucidate the mechanisms of cryoinjury using a fish spermatozoa model. It is known that mitochondrial membrane potential changes occur during cryopreservation, and these changes reflect the functional normality of cryopreserved spermatozoa. Compared to human spermatozoa, fish spermatozoa have lower mitochondrial membrane cryostability. Therefore, fish spermatozoa can be used as a model to investigate cryostability in human spermatozoa damage [15, 16].

### Is vitrification superior to conventional freezing?

Sperm vitrification has been proposed as an alternative after the efforts in past years [7]. Human embryo cryopreservation which has been well established may provide some clues for sperm cryopreservation. Reed et al. [17] reviewed laboratory and clinical data for all frozen embryo replacement cycles from 2012 to 2015 and concluded that vitrification was more effective, as indicated by higher survival in vitrified embryos compared to slow-cooling cryopreservation. Furthermore, vitrification is also reliable and simple to learn and implement in the laboratory while clinical pregnancy and implantation rates outcomes are similar [17], which is supported by other studies have [18–20].

One way to address whether vitrification is superior to conventional freezing is to directly compare the two methods using the same semen samples in the same study (Table 1). With 33 human semen samples, an experiment-controlled study found both slow cryopreservation and vitrification had similar results, but the latter was faster, easier and associated with lower toxicity and cost [28]. Le et al. recently carried out a direct comparison between vitrification and slow cryopreservation [32]. They used 105 human fresh semen samples, exclusive of cryptozoospermia and azoospermia, divided them into washed and unwashed halves, and each group was split into two aliquots: one group cryopreserved by conventional freezing while the other by vitrification. It was demonstrated that conventional freezing resulted in higher motility and viability, while spermatozoa undergoing vitrification were healthier regarding morphology and had fewer defects of sperm head, midpiece and tail [32]. Very recently, Pabon et al. [33] used 47 human sperm samples to compare the efficiency of vitrification and conventional freezing protocols. They found vitrification was optimal for sperm cryopreservation as vitrification protocol resulted in better motility recovery and higher mitochondrial activity [33]. Similar results were reported previously by other researchers [22, 24, 25, 27].

**Table 1:** Comparison of the studies for human spermatozoa conventional freezing and vitrification

Author(s) (year)	Samples recruited	Conventional freezing procedure	Vitrification procedure	Comparison results
Saritha and Bongso [21]	57 human semen samples^a^	Glycerol used. RT 10 min, cryotube 0.85 mL, LN_2_ vapor, 15 + 15 min. Thawed RT 30–45 min.	Glycerol used. RT 10 min. Cryotube 0.85 mL. Plunged into LN_2_. Warmed RT 30–45 min.	No motility difference was found between two methods.
Nawroth et al. [22]	30 human semen samples. Native or swim-up.	Glycerol used. 0.25 mL straw, RT 10 min. 22 °C to 4 °C by − 5 °C/min; 4 °C to − 30 °C by − 10 °C/min; − 30 °C to − 140 °C by − 20 °C/min. Thawed in 37 °C water bath 50 s.	With or without permeable cryoprotectant. Copper loop, 20 μl or 0.25 mL straw. Plunged into LN_2_. Warmed in 37 °C medium 5–10 min.	Permeable cryoprotectant-free vitrification using copper loop resulted in higher motility with swim-up samples than conventional freezing. No difference in morphology.
Chang et al. [23]	30 healthy human semen samples	Freezing medium used. Biological freezer used. Thawing unspecified.	Freezing medium used. Plunged into LN_2_. Warming unspecified.	No difference in motility or DNA fragmentation was found between two methods.
Vutyavanich et al. [24]	30 normospermic human semen samples	Freezing medium used. 0.25 mL straw, RT 10 min. 20 °C to 5 °C by − 1 °C/min; − 5 °C to − 85 °C by − 10 °C/min. Thawed in 25–28 °C tap water.	Glycerol used. 0.25 mL straw, 4 °C 10 min. Plunged into LN_2_. Warmed in 25–28 °C tap water.	Vitrification gave superior motility and cryosurvival than conventional freezing. No difference in morphology or DNA integrity.
Moskovtsev et al. [25]	11 human semen samples. Washed.	Freezing medium used. LN_2_ vapor. Thawing unspecified.	Permeable cryoprotectant free. Warming unspecified.	Vitrification resulted in higher motility and progressive motility.
Agha-Rahimi et al. [26]	30 normozoospermic samples. Washed.	Glycerol used. Cryotube, LN_2_ vapor 30 min. Thawed in 37 °C water bath 10 min.	With or without glycerol. 30 μl drop, Plunged into LN_2_. Warmed at 37 °C medium 5–10 s.	Both methods resulted in similar motility, viability, recovery rate and DNA fragmentatioin. No permeable cryoprotectant was required for vitrification.
Zhu et al. [27]	58 human semen samples. Washed.	Glycerol used. RT 5 min. LN_2_ vapor 30 min. Cryogenic vial, 0.5 mL, LN2 vapor 30 min. Thawed in 37 °C water bath till melted.	Permeable cryoprotectant free. Cryogenic vial, 0.25 mL, RT 1 min. Plunged into LN_2_. Warmed in 42 °C water bath 1 min, 37 °C water bath till melted.	Vitrification with optimal sucrose concentration resulted in higher progressive motility, plasma membrane and acrosome integrity than conventional freezing. No differences in motility or DNA stability.
Ali Mohamed [28]	33human semen samples	Freezing medium used. 0.25 mL straw, RT 10 min, LN_2_ vapor 30 min. Thawed 37 °C water bath till melting.	permeable cryoprotectant free, 37 °C 5 min, 100 μl straw-in-straw, Plunged into LN_2_. Warmed in 42 °C medium.	Both methods had similar motility, viability and mitochondrial membrane potential.
Slabbert et al. [20]	35 human semen samples. Washed.	Freezing medium used. RT 10 min. 0.5 mL straw, LN_2_ vapor 15 min. Thawed in 23 °C 5 min.	permeable cryoprotectant free. 300 μl sample in 1.5 mL straw, RT 10 min. Plunged into LN_2_. Warmed in 42 °C medium 20 s.	Vitrification had higher mitochondrial membrane potential and lower percentage of DNA fragmentation than conventional freezing. No differences in motility.
Tongdee et al. [29]	37 normal human semen samples. Washed.	Freezing medium used. RT 10 min, Cryovial, 0.5 mL, 25 °C to 5 °C by − 1 °C/min; 5 °C to - 85 °C by − 10 °C/min. Thawed RT 15–20 min.	Freezing medium used. RT 10 min, Cryovial, 0.25 mL, plunged into LN_2_. Warmed RT 15–20 min.	Motility decreased more by vitrification. No difference in morphology or DNA intergrity between two methods.
Aizpurua et al. [30]	18 normozoospermic human semen samples.	Glycerol used, 1.8 mL tube, 4 °C 30 min, LN_2_ vapor 30 min. Thawed RT 30 min.	permeable cryoprotectant free, 37 °C 5 min, 20 μl drop, Plunged into LN_2_. Warmed at 37 °C 5 min.	vitrification had higher motility and normal morphology, and lower DNA fragmentation than conventional freezing.
Karthikeyan et al. [31]	20 severe oligoasthenozoospermia (SOA) and very SOA sample^b^	Freezing medium used. 0.5 mL straw, − 85 °C in a deep freezer 1 h. Thawed at RT 10 min.	permeable cryoprotectant free. Cryologic with stripper. Warmed in 37 °C medium	Vitrification revealed higher motility vitality with very SOA samples than conventional freezeing.
Le et al. [32]	105 human semen samples. Washed and unwashed.	Glycerol used. Cryotube RT 10 min. LN_2_ vapor 15 min. Thawed at 37 °C water bath 5 min.	Glycerol used. RT 10 min. 30 μl drop, plunged into LN_2_. Warmed at 37 °C water bath 5 min.	Conventional freezing method resulted in higher motility, viability while vitrification resulted in higher normal morphology.
Pabon et al. [33]	47 human semen samples, Swim-up	Glycerol used. RT 10 min. 50 μl drop on dry ice, cryotube. Thawed RT 10 min, then 37 °C 10 min.	permeable cryoprotectant free. RT 3 min. Collector-grid, 5–10 μl drop, plunged to LN_2_. Warmed in 44 °C medium 3 min.	Vitrification presented higher motility, viability and mitochondrial activity than conventional freezing.
Spis et al. [34]	1 epididymal and 1 testicular sperm samples	Glycerol used. 20 + 8 capillaries. LN_2_ vapor 30 min. Thawed in 37 °C water bath 50 s.	permeable cryoprotectant free. 20 + 8 capillaries. Plunged into LN_2_. Warmed in 37 °C medium 20 s	Vitrification had higher mitochondrial membrane potential and motility in both epididymal and testicular capillaries than conventional freezing^c^.

^a^ 57 human semen samples included 15 normozoospermic unwashed samples, 15 oligozoospermic unwashed, 15 normozoospermic washed samples, 12 oligozoospermic washed

^b^ Very severe oligoasthenozoospermia (VSOA) refers to samples with concentration < 1 million/mL, progressive motility < 10%

^c^ Three healthy babies were born after ICSI using vitrified epididymal (1 baby) and testicular spermatozoa (2 babies)

A recent study recruited 20 subfertile men with semen characteristics of severe oligoasthenozoospermia to compare the effects of two approaches and found that the vitrification method using only nonpermeable cryoprotectants was an effective alternative to the conventional slow-freeze technique [31]. Using epididymal and testicular spermatozoa, Epis et al. also demonstrated that vitrification resulted in higher mitochondrial membrane potential and motility than conventional freezing [34].

In order to compare the effectiveness of two methods, it is also legitimate to review and summarize the previously published and related articles. Recently, a systematic review and meta-analysis was undertaken to compare these methods [35]. The authors reviewed a total of 2428 published articles and 13 randomized controlled trials including 486 vitrified and 486 conventional cryopreserved sperm samples. They concluded that vitrification was superior to conventional freezing in post-thaw motility, including both total motility and progressive motility, although the efficacy of vitrification varied by vitrification protocol and sample quality [35].

Collectively, human sperm vitrification has shown increasing potential although the procedure needs further optimization as discussed later.

### Cryoprotectants and cryodevices in sperm vitrification

Different from embryo vitrification, sperm vitrification with highly concentrated, permeable cryoprotectants is not suitable for spermatozoa because mammalian spermatozoa exposed to hypertonic conditions during cryopreservation results in osmotic shock, and causes the sperm tail to coil at the distal end [36]. Most commonly used permeable cryoprotectants include dimethyl sulfoxide, glycerol, glycol, ethylene and methanol, while albumins, dextrans and egg yolk citrate are often used as nonpermeable cryoprotectants. It is known that controlling the osmolarity during cryopreservation is of great importance. Using permeable cryoprotectants in the cryopreservation media increases the osmolarity, which fluctuates between 600 to 1000 mOsm/L. In contrast, sperm vitrification media is isosmolar between 300 to 396 mOsm/L, which can be achieved with the use of nonpermeable cryoprotectants or a combination. Vitrification of spermatozoa, free of permeable cryoprotectants, is important to maintain sperm fertilization potential such as capacitation, acrosome reaction, and the integrity of cytoplasmic and mitochondrial membranes. Indeed, unlike the results noted above, using permeable cryoprotectants in sperm vitrification resulted in compromised or even lower motility than conventional freezing with permeable cryoprotectants [21–23, 29] (Table 1). Isachenko et al. [18, 19] used 52 human swim up-prepared ejaculates for vitrification without permeable cryoprotectant. They found that in contrast to conventional freezing with glycerol, vitrified sperm displayed higher motility and integrity rates of cytoplasmic and acrosomal membranes, while no difference in spontaneous cryo-capacitation or acrosome reaction was observed [18].

Slabbert et al. [20] used 35 human semen samples for permeable cryoprotectant-free vitrification. They found that the cryoprotectant-free technology generated higher mitochondrial membrane potential but lower DNA fragmentation with no difference in post-thaw motility compared to conventional slow freezing [20].

Aizpurua et al. [30] used 18 normozoospermic sperm samples to test a permeable cryoprotectant-free vitrification protocol. They found that it generated better recovery rates of good quality sperm and better maintenance of sperm quality than traditional slow-freezing [30]. In addition, they also demonstrated that permeable cryoprotectant-free vitrification presented a higher percentage of live spermatozoa, better preservation of acrosomes, and lower DNA fragmentation. Furthermore, using α-tubulin immunocytochemistry to show sperm cytoskeleton, they found that vitrified sperm had similar labeling patterns in the tail to fresh sperm, but different from slow freezing [30]. However, Agha-Rahimi et al. [26] vitrified 30 human normospermic samples with and without cryoprotectant, and they found that sperm frozen in permeable cryoprotectant did not show any toxicity or any difference in post-thaw motility, DNA fragmentation, or hyaluronan-binding potential [26].

In terms of nonpermeable cryoprotectants, some might perform better than others at specific concentrations. For instance, Schulz et al. compared two different nonpermeable cryoprotectants in vitrification with healthy volunteer semen samples and found post-warming sperm motility using 0.1 mol/L trehalose (69%) was higher than that of widely used 0.25 mol/L sucrose (58%). Furthermore, similar results were obtained at 6 and 12 h post-thaw. 0.1 mol/L trehalose preserved sperm had improved membrane integrity at 0 h post thaw, although no significant improvements at 6 h or 12 h compared to sucrose [37]. Another study found butylhydroxytoluene (BHT), a synthetic analogue of vitamin E, effectively maintained vitrified sperm function at 1 mmol/L, including higher progressive sperm motility after warming, DNA integrity, and lower reactive oxygen species [38]. Further research is needed to investigate the optimal nonpermeable cryoprotectants, their concentrations, or combinations depending on different human semen parameters or on clinical purposes.

Besides open systems like cryoloop, a number of closed carriers have been developed and applied in vitrification, including straw-in-straw, high security vitrification straw, Cryotip, VitriSafe, Cryopette, cryoLogic, Rapid-i, S3 system, and the S3 μS-VTF device [39–42], many of which have proven effective for different purposes. More carriers are expected to be developed and optimized to facilitate sperm vitrification in the future. With the rapid progress of 3-D printing technology, it is now possible to design and print a freezing device. This technique could become a flexible, inexpensive and standard approach to produce freezing devices [43]. One of the 3-D printing thermoplastics, polylactic acid, has proved to be well suited to use for cryogenic activities and is routinely used in 3-D printers already. Because engineering grade 3-D printing can be easily standardized and modified, 3-D printing to form a freezing device has shown its potential applications in sperm cryopreservation.

### Does vitrification have to be ultra-fast freezing?

It is believed that, to avoid cryo-damage in vitrification, the loading volume of samples in each carrier must be low enough to achieve high cooling rates [44, 45]. Reducing volume also helps reduce the chance of ice nucleation. By direct plunging into LN_2_, sperm samples can reach cooling rates of 2, 000–10, 000 °C/min. However, reducing sample volumes limits efficiency in practice, since too many frozen units with small volumes (≤20 μl) not only make the operation process time-consuming, but also make it hard to collect enough number of motile sperm after warming.

[30] compared the efficiency of slow and ultra-rapid freezing and determined the level of DNA fragmentation after slow freezing-thawing and vitrification-warming. With 18 normal human semen samples, they found that ultra-rapid freezing resulted in higher progressive motility (18% vs 11%), higher mormal morphology rate, vitality, and lower sperm DNA fragmentation (20% vs 27%) compared to slow freezing. They concluded that the sperm ultra-rapid freezing was superior to slow freezing [30]. More recently, Hosseini et al. [46] prepared human samples from normozoospermic ejaculates by swim-up, and low number of human spermatozoa were frozen by directly submerging in LN_2_ or its vapor. They found that direct submerging generated higher sperm progressive motility and total motility rates, lower alterations in sperm chromatin indicated by chromomycin-A3 and Aniline Blue staining, but did not affect morphology, acrosome integrity or DNA damage [46].

Using different size of straws for vitrification, a research group vitrified 22 fertile human donor semen samples with few spermatozoa. They used a micro-straw (50–100 μl) as well as traditional straws (0.25 ml and 0.5 ml). Compared to sperm samples frozen in traditional straws, the sperm frozen in micro-straws showed higher sperm motility after freezing-thawing with no difference in morphology, acrosome, or DNA integrity [47]. The authors thought as the micro-straws were thinner and they hold very small volume, the freezing rate was much faster. To freeze a very small number of human spermatozoa, a multi-well mini plate has been newly developed for vitrification using ~ 1 μl droplets. Sperm frozen using this method showed descent recovery rates and post thaw motility [48]. This novel approach was commented by Paffoni and Palini [49] [49].

As noted above, some scientists suggested using fish spermatozoa freezing model to investigate the mechanisms of why cryoprotectant-free vitrification for human ejaculates is better than conventional freezing and vitrification with cryoprotectants [15, 16, 50].

In contrast, Isachenko et al. [51] found the cooling speed didn’t have to be ultra-fast. They compared the quality of human sperm vitrification method (720, 000 °C/min) and relative slow cooling using LN_2_ vapor (150–250 °C/min) without any cryoprotectant agents by cryoloop. They found both cooling modes led to comparable results in terms of the motility, fertilization ability, and DNA integrity of the warmed spermatozoa [51]. It appears that a wide range of cooling rates are applicable. If the cooling speed during vitrification is flexible, aseptic vitrification with double straw containing higher volumes of 0.1 to 0.5 mL would allow the use of these techniques in human ART [18, 19]. Interestingly, in human embryo vitrification, embryo survival is higher for large-volume vitrification, which has lower cooling rate comapred to micro-volume vitrification [17].

### Vitrified specimen warming

Besides the high-speed freezing (2, 000 °C/mL), the warming velocity should also be high so that the water inside spermatozoa passes from glassy state to liquid without ice crystal formation. Using human spermatozoa samples, Sanchez et al. [52] found the temperatures in the devitrification process were essential for preserved morphological membrane integrity and sperm function [52]. Furthermore, Mansilla et al. [53] tried to determine the optimal warming temperature after human spermatozoa vitrification and found the progressive motility in sperm samples warmed at 42 °C (65%) was higher than those at 38 °C (26%) and 40 °C (57%) and plasma membrane function was also better preserved at 42 °C [53]. Ali Mohamed [28] also warmed vitrified human sperm samples in prewarmed medium at 42 °C [28]. Pabon et al. warmed vitrified human spermatozoa micropills (5–10 μl each) in 500 μl medium prewarmed and maintained at 44 °C for 5 s and observed descent post-thaw motility and mitochondrial activity [33]. Another report noted above warmed vitrified human sperm samples (30 μl pellets) in a 37 °C water bath for 5 min resulting in motile and fertile sperm [32], indicating the flexibility of warming procedure.

## What is the contamination risk using sperm vitrification?

Most of the proposed sperm vitrification methods, such as cryoloop, are described as open systems to obtain high cooling rate [51]. This brings the major disadvantage of potential contamination risk as open systems expose the semen samples directly to LN_2_.

Nitrogen was first liquefied in 1883 by Polish physicists Wroblewski and Olszewski. The liquid-to-gas expansion ratio of nitrogen is 1:694. Liquid nitrogen is commercially produced by compressing air and using fractional distillation. Unless specifically requested, commercial liquid nitrogen provided by suppliers is not sterile. Therefore, it is a potential source of contamination as a number of infectious agents can survive at cryogenic temperatures. It has been well documented that unsterilized commercial LN_2_ possibly carries microorganisms which increase the risk of transmission and propagation of diseases. A large variety of bacterial, viral and fungal species has been found in liquid nitrogen [54–57]. Common bacterial contaminants include *Pseudomonas* spp.*, Enterobacter cloacae, Staphylococcus sciuri, Acinetobacter calcoaceticus,* and *Flavobacterium* spp. [54].

Indeed, the survival of cryogenic pathogens in LN_2_ generates the possibility of cross-contamination between LN_2_ and stored samples because sterile LN_2_ can get contaminated from stored contaminated semen samples thus become a source of contamination itself [50, 54, 58]. The ingredients used during cryopreservation, especially the cryoprotectants and media increase the survivability of these pathogens. Furthermore, repeated freezing and warming could affect the survival of pathogens in different ways [59]. Relatively, fungi are sensitive to freezing while bacteria have a high tolerance. Piasecka-Serafin [60] reported translocation of bacteria from infected semen pellets, to sterile LN_2_, and then to sterile semen pellets. Within only 2 hours of cryostorage, as many as 94% of the sterile samples became contaminated with *E. coli* and *S. aureus* [60]. The ability of pathogens to survive in LN_2_ was further demonstrated in LN_2_ contaminated with infectious vesicular stomatitis virus [61] and then cross contamination with hepatitis B from cryostored bone marrow due to a packaging leak. This leak affected 4 patients receiving cryostored bone marrow [62].

The ability of cryopreserved materials to become contaminated after storage in contaminated LN_2_ has been shown in LN_2_ purposely spiked with different viruses. In this study, bovine embryos were vitrified in either unsealed standard 0.25 ml or modified open pulled straws or in sealed plastic cryovials and then plunged into contaminated liquid phase nitrogen. After 3–5 weeks of storage in the contaminated LN_2_, the bovine embryos were thawed and sequentially washed. Only those with intact zona pellucida (ZP) were pooled together and tested for bovine viral diarrhea virus (BVDV), bovine herpesvirus-1 (BHV-1), and bovine immunodeficiency virus (BIV). The results showed that all control embryos vitrified in sealed cryovials and straws were free from any viral contamination, but 13 of 61 (21%) batches exposed to BVDV and BHV-1 were positive for viral association while none of 22 batches exposed to BIV in unsealed containers was BIV-positive [54, 56].

Surprisingly, Cobo et al. [63] screened the culture medium and LN_2_ used to vitrify oocytes and embryos of 24 women for viral RNA and DNA. They found that none of 33 used culture media samples or 27 used LN_2_ samples was positive with any HIV, hepatitis B virus (HBV) or hepatitis C virus (HCV) contamination, even using an open device for vitrification [63]. A total of 6, 11, and 6 patients were seropositive for HIV, HCV, and HBV, respectively, whereas 1 patient showed a coinfection with HCV and HBV. Seven patients presented positive blood viral load (1 HIV, 1 HBV, 5 HCV). The results of this study may be limited due to the relatively low sample size.

Molina et al. [64] directly compared the contamination risk of bacteria and fungi between open and closed vitrification devices with human oocytes and embryos. Interestingly, they also found that the bacteria cross-contamination risk was no greater for open containers than for closed containers in vitrification and no bacterial or fungal contamination was observed in either open or closed devices storing human oocytes and embryos after 1–2 years storage [64]. To identify the bacteria, suspected samples were inoculated on different agar plates and cultured 2–3 days. If any colonies were presented following innoculation, each was sub-cultured to a new plate for purity. Pure cultures were then Gram stained for morphology and identified. Fungal detection was carried out on Sabouraud dextrose agar with chloranphenicol and the filamentous fungi were identified on the basis of macroscopic and microscopic morphologic features. They found all the five containers used to store oocytes and embryos for 1–17 years were contaminated with bacteria, mainly *Bacillus spp*, *Stenotrophomonas maltophilia* and *Enterobacter spp*., before and after LN_2_ filling, but none with fungi although the media, device or the LN_2_ used were free from either bacteria or fungi before use. There were *Acinetobacter lwoffii*, *Alcaligenes faecalis ssp. faecalis*, and *Sphingomonas paucimobilis* at the bottom of storage containers but no fungi were observed. The contamination had no correlation to the number of samples stored or to the time the container had been used. The source of these pathogens could be the cryopreservation environment [64].

Joaquim et al. [58] reviewed the potential hazard of infectious transmission through cryopreserved and banked gametes and embryos in LN_2_ during cryopreservation including viruses, bacteria and fungi [58]. In recent years, Zika vius has been widespread, and known to cause a *serious* birth defect known as microcephaly. It is still unknown if Zika virus is able to survive in LN_2_, but the British Fertility Society had suggested the possibility [65, 66].

### Contamination control

No freezing method is absolutely safe. Frequent cleaning of used utensils should be the basic measure, including dry shippers, tanks, dewars, canisters, canes, and sample carriers. However, such maintenance may require samples to be removed from storage, which could put the stored specimen at risk [54–56, 58, 67]. Another basic rule to avoid contamination is to store contaminated samples separately in quarantine to minimize the risk of cross contamination if possible. Commercially produced LN_2_ itself could contain cryogenic pathogens and become the source of contamination. However, obtaining a small amount of sterile LN_2_ is feasible by sterilizing the air used to create LN_2_. For instance, McBurnie and Bardo [68] demonstrated that air filtration with 0.22 μm polytetrafluoroethylene efficiently retained *Brevundimonas diminuta* with extreme temperatures, high pressures, high flow rates, and high concentration of bacteria before LN_2_ manufacturing. Alternatively, filtration of regular but non-sterile LN_2_ before the samples are exposed could be even simpler. Indeed, a device called CLAir was developed for use in vitrification of human oocytes and mouse embryos [69]. A 0.22-μm filter equipped inside the canister can produce sterile liquid air at similar temperature to LN_2_ so that the samples saved in a sealed canister (esther) are only exposed to sterile liquid air. Liquid air showed the same vitrification outcome with human oocytes and mouse embryos but with no contamination while large amounts of contamination with regular LN_2_ were observed. Presumably, such device could be used to prevent contamination in the sperm vitrification process.

Another basic strategy to control contamination is to avoid direct contact with LN_2_. It is therefore understandable that closed carriers as used in conventional freezing show lower incidence of contamination in comparison to open ones. Cryoloop, a widely used device in which the specimen is directly submerged into LN_2_, generates considerable vitrification effects at the expense of severe sample contamination [55]. As using a closed carrier in the vitrification process is not always feasible, evidence has shown that using liquid nitrogen vapor, instead of LN_2_ itself, to store human sperm samples can lower the risk of viral cross-contamination [70]. Fortunately, efforts to improve the use of closed carriers in sperm vitrification have been made and have shown encouraging results. For example, Isachenko et al. [18, 19] developed an aseptic technology for human spermatozoa vitrification. They used in 0.5 mL insemination straws for immediate intrauterine insemination and achieved satisfactory outcome with normozoospermic and severely oligozoospermic samples [18]. Slabbert et al. [20] used 0.5 ml straws to load 300 μl sample to allow sufficient air space inside the straw to prevent rupturing when immersed into LN_2_. This method worked successfully with 35 vitrified human semen samples [20]. Diaz-Jimenez et al. [71] cryopreserved six donkey ejaculates, which were vitrified with either 30 μl sperm solution sphere suspensions or 100 μl in 0.25 ml straws with 0.1 M sucrose without glycerol. They found the straw method resulted in higher total and progressive motility while no difference in plasma membrane integrity [71]. It appears that sperm vitrification does not compromise post-thaw motility when using a closed carrier. Straw-in-straw design, or double straw, allows the inner straw contain the specimen and then it is sealed and inserted to outer straw, and then the whole unit is submerged to LN_2_ so there is no direct exposure to LN_2_. The design showed considerable vitrification results with 82 mouse D2/D3 embryos in 30 μl solution [72] and 113 human oocytes and 93 blastocysts using 1 μl medium [73]. Still, when loaded, the inner straw containing specimen could explode during freezing and warming due to the air pressure fluctuations. Therefore, a thin, narrow-walled capillary was developed to speed up the temperature conduction and reduce the air volume [74–76]. The contamination incidence of this double straw method should be no greater than that of conventional method.

Intermediate methods exist that are a hybrid of open and closed systems to achieve the benefits of each method. Samples can be vitrified by direct contact to a small amount of purified LN_2_, and are then sealed and stored in large quantities regular LN_2_. This method has been reported in human embryo vitrification [77], and could be tested for efficacy in sperm vitrification.

Ultraviolet (UV) light could also be a possible solution to reduce the contamination risk to vitrified sperm samples. Treating a small volume of LN_2_ with UV light at a suitable dose of radiation has been demonstrated to effectively reduce the number of pathogens, including bacteria, viruses, and fungi [78]. It was reported that 8, 000 μW/cm^2^ of UV light could destroy hepatitis B virus while 330, 000 μW/cm2 destroyed the fungus *Aspergillus niger*. Most virus become inactivated by UV light at dose of 200, 000 μW/cm^2^ [79] while Zika virus may have higher resistance to UV light [79]. Therefore, UV light could be a viable solution to reducing contamination rates in non-sterile LN_2_.

Unfortunately, the application of UV light to LN_2_ containing human samples is controversial. The UV light used could also cause severe genetic aberrations to stored spermatozoa, and further to fertilized embryos, although a study with human oocytes has demonstrated no adverse effects [80]. A simple solution is to sterilize LN_2_ with UV light before it is used to freeze and store semen samples. Rinsing contaminated samples with sterile LN_2_ can significantly reduce the contaminant pathogens. Parmegiani et al. [75] washed human oocytes and embryos purposely contaminated with bacteria (*P. aeruginosa*, *E. coli,* and *S. maltophilia*) and a fungus *(A. Niger)* three times in LN_2_ sterilized by UV light. Washing these samples in sterilized LN_2_ eliminated the contamination of both bacteria (0/65) and the fungus (0/25) while the unwashed samples remained highly contaminated with both bacteria (92/117) and fungi (25/25) [75]. Another concern with the use of UV light to sterilize LN_2_ is the generation of ozone, which could have detrimental effects on the buffering system in which the cryogenic samples are stored. Luckily, the formation of ozone from UV light is insignificant as ozone is formed by the breakdown of oxygen molecules by the action of UV radiation. When these oxygen atoms separate, they combine with other oxygen molecules to form ozone. However, as LN_2_ is virtually free from oxygen, this should not be an issue with UV light sterilization of LN_2_ [80].

Generally, there is no easy but cost-effective way to completely eliminate all the potential risks of contamination in sperm vitrification. But based on the improvements in past years, it is possible to control the contamination risk of vitrification to the level of conventional freezing.

### Is there a universal vitrification protocol for all types of sperm samples?

Careful optimization of preservation protocols can be tedious, confusing and expensive due to the specific devices and reagents required. Is it possible to develop a universal protocol for all the species and all the samples? It sounds impossible since the cryotolerance of spermatozoa depends on sperm features such as size, shape, and lipid composition, which makes it challenging to generate a single standardized freezing procedure for all species. Even in humans, there is a variety of sperm specimens, such as normospermic, oligospermic, azoospermic samples, testicular sperm aspiration (TESA), and percutaneous epididymal sperm aspiration (PESA) samples with different parameters such as volume, concentration, motility, and seminal plasma features. Furthermore, it is difficult to establish a universal stereotyped model to serve different cryopreservation purposes at human clinic. Rozati et al. [81] reviewed the pitfalls of human sperm cryopreservation, especially the sperm banking for cancer patients [81].

In fact, there was an effort to establish a universal vitrification method for almost all the samples including oocytes, primary cells, stem cells, and genetically modified cells. The method proposed low concentrations of cryoprotectants including 1.5 M propanediol and 0.5 M trehalose in industrial grade microcapillaries made of highly conductive fused silica. It was demonstrated that this universal protocol achieved high recovery and viability rates after vitrification for human mammary epithelial cells, rat hepatocytes, tumor cells from pleural effusions, and multiple cancer cell lines [82]. Unfortunately, due to the different characteristics of spermatozoa from the cell types tested, this method would not likely be superior to the specialized sperm cryopreservation protocols that exist presently.

## Conclusion

In the past decade, clinical application of vitrification was one of the most important achievements in human ART. Currently, conventional sperm freezing is still the primary method used for sperm cryopreservation at ART clinics, but sperm vitrification has shown great advantages. Technically, sperm vitrification has substantial difference from conventional slow freezing and from oocyte and embryo vitrification due to the propensity of sperm to become cryodamaged. More and more studies have shown that a vitrification approach free from any non-permeable cryoprotectants performs better than conventional slow freezing protocol for human sperm cryopreseravtion. Sperm vitrification usually requires a small volume of load to achieve high cooling rate, which makes it less feasible with samples of large volumes, but in the past few years, many new designs of larger volume have been tested and developed, and promising results have been achieved. The potential risk of contamination from using open carriers to obtain ultra-fast freezing speeds has been a concern of sperm vitrification. Recently, more and more strategies have been raised to reduce the contamination risk, including new vitrification carriers/designs to both control the risk and improve cryopreservation efficiency. The advancements of 3-D printing tested in recent years could provide a novel approach for manufacturing freezing devices that minimize contamination risk. In terms of potential contamination risk to a variety of cryogenic pathogens, many possible solutions have been identified to control and reduce contamination risk to acceptable levels. Finally, depending on the variety of semen parameters and on the personalized purposes at ART clinics, specific sperm cryopreservation approaches should be individually designed to reach the optimal results.

## Data Availability

N/A

## Abbreviations

3-D: 3 dimensional
ART: Assisted reproductive technology
BHT: Butylhydroxytoluene
BHV-1: Bovine herpesvirus-1
BIV: Bovine immunodeficiency virus
BVDV: Bovine viral diarrhea virus
HBV: Hepatitis B virus
HCV: Hepatitis C virus
HIV: Human immunodeficiency virus
ICSI: Intracytoplasmic sperm injection
LN_2_: Liquid nitrogen
PESA: Percutaneous epididymal sperm aspiration
TESA: Testicular sperm aspiration
UV: Ultraviolet
ZP: Zona pellucida
